# Pathologic Complete Response (pCR) and Survival of Women with Inflammatory Breast Cancer (IBC): An Analysis Based on Biologic Subtypes and Demographic Characteristics

**DOI:** 10.3390/ijerph16010124

**Published:** 2019-01-04

**Authors:** Tithi Biswas, Charulata Jindal, Timothy L. Fitzgerald, Jimmy T. Efird

**Affiliations:** 1 Department of Radiation Oncology, University Hospitals, Case Western Reserve University, Cleveland, OH 44106, USA; tithi.biswas@uhhospitals.org; 2 Centre for Clinical Epidemiology and Biostatistics (CCEB), School of Medicine and Public Health, The University of Newcastle (UoN), Newcastle 2308, Australia; charu.jindal@uon.edu.au; 3 Surgical Oncology, Maine Medical Center Cancer Institute, Scarborough, ME 04074, USA; TLFitzgera@mmc.org; 4 Priority Research Centre for Generational Health and Ageing (PRCGHA), School of Medicine and Public Health, The University of Newcastle (UoN), Newcastle 2308, Australia

**Keywords:** biologic subtypes, diagnosis, inflammatory breast cancer, pCR, survival

## Abstract

In this US-based study of the National Cancer Database (NCDB), we examined 8550 patients diagnosed with non-metastatic, invasive inflammatory breast cancer (IBC) who received surgery from 2004–2013. Patients were grouped into four biologic subtypes (HR^+^/HER_2^−^_, HR^+^/HER_2^+^_, HR^−^/HER_2^+^_, HR^−^/HER_2^−^_). On average, women were 56 years of age at diagnosis and were followed for a median of 3.7 years. The majority were white (80%), had private health insurance (50%), and presented with poorly differentiated tumors (57%). Approximately 46% of the cancers were >5 cm. Most patients underwent mastectomy (94%) and received radiotherapy (71%). Differences by biologic subtypes were observed for grade, lymph node invasion, race, and tumor size (*p* < 0.0001). Patients experiencing pathologic complete response (pCR, 12%) vs. non-pCR had superior 5-year overall survival (OS) (77% vs. 54%) (*p* < 0.0001). Survival was poor for triple-negative (TN) tumors (37%) vs. other biologic subtypes (60%) (*p* < 0.0001). On multivariable analysis, TN-IBC, positive margins, and not receiving either chemotherapy, hormonal therapy or radiotherapy were independently associated with poor 5-year survival (*p* < 0.0001). In this analysis of IBC, categorized by biologic subtypes, we observed significant differential tumor, patient and treatment characteristics, and OS.

## 1. Introduction

Inflammatory breast cancer (IBC) is an aggressive breast cancer with rapid onset and poor outcomes [1,2]. According to the Surveillance, Epidemiology, and End Results (SEER) registry, its population-based incidence is approximately 1.3% [3]. However, this figure varies widely, from <1% to 10%, depending upon different case definitions reported in the literature [4,5,6,7,8,9,10,11,12,13]. Originally described by Sir Charles Bell in 1814, IBC has been recognized by its distinct clinical characteristics. This includes rapid onset of breast skin erythema with edema (known as peau d’orange) [14,15,16].

The classic appearance of IBC is attributed to tumor emboli invasion of the dermal lymphatic vessels, which may or may not be seen on skin biopsy [17]. The diagnosis of IBC is by its clinical appearance and/or pathologic features, with the latter not being required to confirm the diagnosis [5,18]. IBC is characterized by either diffused or localized radiographic density [19]. By convention, the signs and symptoms of IBC appear within a 6-month period following first indication [20]. Overall, the 5-year survival for IBC remains poor (55% among patients receiving triple-modality therapy) [21].

Analogous to non-IBC, IBC has five molecular subtypes based on their gene expression profile: luminal A, luminal B, human epidermal growth factor receptor 2 (HER_2_) over-expression, basal, and normal-breast like [22,23]. Furthermore, IBC can be characterized according to phenotypic expression of estrogen/progesterone hormone receptors (HR) and HER_2_ and often is grouped into 4 distinct biologic subtypes (HR^+^/HER_2^−^_, HR^+^/HER_2^+^_, HR^−^/HER_2^+^_, HR^−^/HER_2^−^_) [24]. HR^−^ status implies that either estrogen and/or progesterone receptors are absent on the tumor cell surface. 

Pathologic complete response (pCR) refers to the absence of invasive/in situ cancer in the breast and/or axillary lymph nodes [25,26,27]. Achieving pCR following neoadjuvant chemotherapy (NACT) is a desirable outcome, frequently leading to improved survival [28,29]. This is especially relevant in the modern era with the introduction of HER_2_ targeted therapies and the more frequent use of anthracyclin + taxanes vs. cyclophosphamide/methotrexate/5-fluorouracil (CMF) [30,31,32,33,34]. However, the extent of this benefit appears to vary by biologic subtypes [26]. In contrast to breast cancer as a whole, IBC is a comparatively rare malignancy and has not been well characterized by biologic subtypes, pCR and associated outcomes [35,36]. We undertook this study to examine pCR and OS of IBC, based on different biologic subtypes. 

## 2. Materials and Methods 

### 2.1. Data Source

The NCDB database has been previously described [37,38]. In brief, over 1500 Commission on Cancer (CoC) accredited cancer programs in the United States (US) report data to the NCDB, encompassing approximately 70% of incident cancer cases [39]. The database is the largest cancer registry in the world and contains nearly 10 million cases. In comparison, only 25% of new cancer cases are identified through the SEER program [40]. Participant hospitals must satisfy 35 standards pertaining to the delivery of cancer care in order to be accredited by CoC. Every three years, hospitals are re-evaluated for their compliance with these standards. Records in NCDB are de-identified. NCDB has been collecting information on biologic subtypes for breast cancer since 2004. This study was considered exempt by the institutional review board (IRB) at the recipient NCDB member facility (Code of Federal Regulations 45 part 46.101(b)).

### 2.2. Eligibility

Patients with primary histologic diagnosis of invasive ductal, lobular or other primary breast histology subtypes undergoing any surgical resection from 2004–2013 were included in the analysis dataset. Patients were excluded if their radiotherapy (RT) dose was not within the range of 4000–6000 centigray (cGy) or the primary target was outside the breast, chest wall or lymph nodes. Sarcomas, lymphomas, leukaemias, and in situ tumors of the breast also were excluded in the analyses.

### 2.3. Definitions

Clinical and pathological stage were coded and assessed by each CoC facility based upon the American Joint Committee on Cancer (AJCC) Tumor, Nodes and Metastasis (TNM) system [41]. The majority of patients were staged according to the sixth and seventh editions of this coding system. Data was not converted from the lower TNM editions. Instead, a sensitivity analysis was performed by stratifying the data by year of diagnosis, with the cut-off value based on the year that the seventh edition was introduced (i.e., 2010). 

Comorbidities were categorized using the Charlson (Deyo) Comorbidity Index (CCI). In this index, comorbid conditions are mapped using up to 10 International Classification of Diseases, 9th Revision, Clinical Modification (ICD-9-CM) secondary diagnosis codes and assigned a weighted score between 0 and 25. Patients with no comorbidities were assigned a score of 0. In NCDB, the highest score of 2 is a truncated value corresponding to the presence of multiple comorbidities [42,43]. The surgical procedure of the primary site was dichotomized based on NCDB surgical codes as breast conserving surgery (BCS)/partial mastectomy (20–24) and mastectomy (30, 40–46, 50–56, 60–67, 70–72, 80).

IBC is primarily a clinical diagnosis, with essential pathologic confirmation of invasive carcinoma [44]. Classification does not require pathologic evidence of tumor emboli in the dermal lymphatics. Radiologic signs for IBC also are insufficient as a diagnostic criteria. In our study, IBC was defined as AJCC clinical stage IIIb/c tumors that were either: (1) clinical/pathology stage T4D, (2) histology code 8530, or (3) had a site specific extension code indicative of IBC (518, 519, 520, 575, 600, 613, 615, 620, 710, 720, 715, 725, 730, 750, 780). Tumors coded as pT4D specifically required clinical features to be present (e.g., diffuse erythema and edema involving a third or more of the skin of the breast).

Response to NACT was recorded in the NCDB database as collaborative stage site-specific factor 21 (CSF-21), based on clinician documentation. The great-circle (orthodromic) distance referred to the shortest distance between a patient’s place of residence and the treating facility. Tumor size at diagnosis was determined radiologically, using the maximum measurement if more than one dimension was available.

### 2.4. Statistical Analysis

Categorical variables were denoted as frequency and percentage, while continuous variables were reported as median and interquartile range (IQR). Statistical significance for categorical variables was tested using the chi-square (χ^2^) procedure and the Kruskal-Wallis H test for continuous variables. A proportional hazard model was used to analyze 5-year survival, with corresponding probabilities computed using the Kaplan-Meier (product-limit) method. Follow-up time was measured from the date of diagnosis (baseline) to death (or censoring at 5 years). Variables with HR ≥ 2.0 (or ≤0.50) and *p* < 0.0001 in univariable analysis were included in the multivariable Cox regression survival model. The method of Grambsch and Therneau was used to test the proportional-hazards assumption of our survival models [45].

Unless indicated otherwise, the reference group for binary coded variables was the complement of the indicated category. Other variables were categorized according to NCDB definitions. A multistage expectation-maximization (EM) algorithm was used to handle missing values [46]. Statistical significance was defined as *p* ≤ 0.05. SAS statistical software (version 9.4, SAS Institute Inc., Cary, NC, USA) was used for all analyses.

## 3. Results

The median age of women at diagnosis was 56 years (*n* = 8550; IQR = 18) (Table 1). On average, they were followed for a median of 3.7 years [IQR = 4.3]. Over half of the patients had private health insurance and lived more than nine miles from their treatment facility, which in most cases was a comprehensive community cancer center (47%). White race was the predominant group within each biologic subtype (≥80%). Less than 4% of patients were classified as “other race”. Approximately 8% of the sample were Hispanic ethnicity. Independent of biologic subtype, over 80% of our patients presented with no significant comorbidities (CCI score = 0). 

A total of 3692 (43%) of patients presented with HER_2^+^_ tumors. HR^+^/HER_2^−^_ (46%) and HR^+^/HER_2^+^_ (36%) were the most common biologic subtypes, followed by HR^−^/HER_2^−^_ (10%) and HR^−^/HER_2^+^_ (7%). Triple negative-IBC (TN-IBC) (25%) was the most frequently occurring subtype among black patients, with HR^+^/HER_2^−^_ having the lowest representation (14%).

The majority of patients presented with clinical stage IIIb disease (82%) and had poorly differentiated tumors (57%) (Table 2). TN-IBC had the highest percentage of grade III tumors (75%). For increasing tumor size at diagnosis, the percentages of patients within each category increased in a corresponding fashion (≤2 cm, 12%; >2–5 cm, 43%; >5 cm, 46%). The lowest risk of lymph node invasion was for HR^−^/HER_2^+^_ (86%) compared with other biologic subtypes. Positive margins were present in 13% of the patients. A total of 4940 (58%) presented with ‘infiltrating duct carcinoma (not otherwise specified)’ (ICD-O-3 = 8500) (not shown in tables). Other histologic subtypes included ‘infiltrating/invasive lobular carcinoma’ (ICD-O-3 = 8520, 8522) (*n* = 578; 7%), ‘infiltrating duct mixed’ (ICD-O-3 = 8523) (*n* = 135; 2%) and ‘other’ (*n* = 2897; 34%).

Approximately 71% of patients received radiotherapy, with a median dose of 5040 cGy (IQR = 40) (Table 3). Of this group, 70% received regional node irradiation (RNI). Systemic chemotherapy (neoadjuvant and/or adjuvant) was administered to over 90% of patients, while ~50–60% of patients with HR^+^ IBC received endocrine therapy. Patients with HR^−^/HER_2^+^_ and TN subtypes were more commonly given NACT than those with positive hormone receptor status. Neoadjuvant endocrine therapy was used in 15% patients (not shown in tables). The greatest pCR rate was observed for women with HR^−^/HER_2^+^_ tumors (27%) (*p* < 0.0001).

Mastectomy was the primary modality of surgery with partial mastectomy being used in only 5–6% of patients. Less than 25% of women underwent contralateral mastectomy, which may have been prophylactic or for bilateral disease. 

On univariable analysis, chemotherapy (HR = 0.41), hormone therapy (HR = 0.46), and radiotherapy (HR = 0.47) were associated with improved survival (*p* < 0.0001), while TN-IBC (HR = 2.2) and positive margins (HR = 2.0) conferred poorer survival (*p* < 0.0001). 

**Figure 1:**
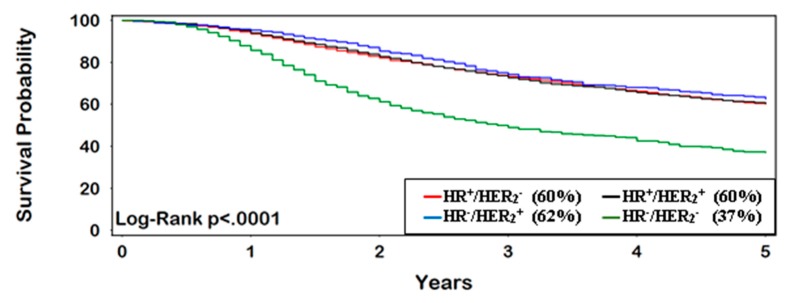
All patients (5-year survival).

**Figure 2:**
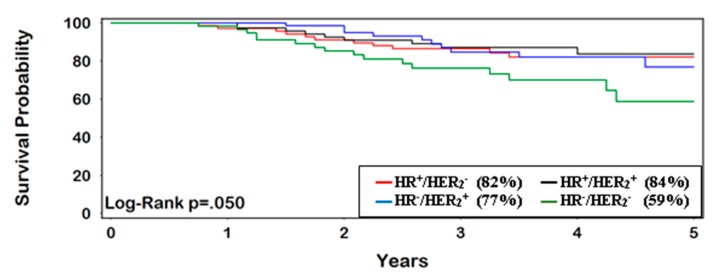
Patients with pCR following neoadjuvant therapy (5-year survival).

**Figure 3:**
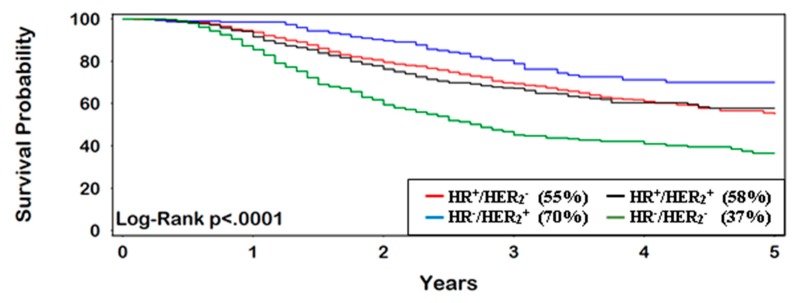
Patients without pCR following neoadjuvant therapy (5-year survival).

OS for patients with IBC at 5 years was 58% (95% CI 57–59%). TN-IBC had the lowest survival rate, with only 37% (95% CI 33–41%) surviving 5 years, compared with other subtypes (*p* < 0.0001) (Figure 1). Women who achieved pCR had consistently better OS at 5 years (77%, 95% CI 70–83%) vs. non-pCR (54%, 95% CI 51–56%) (HR = 0.40, 95% CI 0.29–0.53) (Figure 2 and Figure 3). The greatest improvement in 5-year survival following pCR (compared with non-pCR) was for TN-IBC.

On multivariable analysis, TN-IBC subtype, positive margins and grade III/IV tumors were significant predictors of poor 5-year OS (*p* < 0.0001) (Table 4). Any systemic therapy and radiotherapy were associated with improved OS (*p* < 0.0001). Further pairwise adjustment for age, clinical stage, comorbidities, insurance type, grade, great circle distance, Hispanic ethnicity, immunotherapy, income, insurance status, lymph node invasion, lumpectomy, NACT, race, and tumor size did not substantively impact the model.

In a separate multivariate analysis adjusting for the factors shown in Table 4, black race (referent = non-black) was associated with shorter survival (HR = 1.3, 95% CI 1.2–1.4), while Hispanic ethnicity (referent = non-Hispanic) was associated with longer survival (HR = 0.81, 95% CI 0.71–0.92).

## 4. Discussion

### 4.1. Phenotypic Subtypes of IBC

The expression of different cell growth and apoptosis related markers on the surface of breast cancer cells play an important role in disease prognosis and management. To aid in decision-making, breast cancer is typically classified into biologic subtypes based on their phenotypic expression of HR and HER_2_ receptors [35,47]. However, while phenotypic subtypes are important for predicting outcomes among women with non-IBC, this is not well established for IBC.

### 4.2. Treatment

All patients in our study underwent surgical resection, often as part of a trimodal approach including chemotherapy and radiotherapy. Depending upon tumor characteristics, primary systemic therapy was administered as a means to downstage the tumor and a necessary component of systemic disease management. NACT typically consisted of anthracycline based poly-chemotherapy and Trastuzumab (in the case of HER_2^+^_ tumors). Neoadjuvant or adjuvant hormone therapy when applicable was administered to patients with HR^+^ tumors. Additionally, trastuzumab (Herceptin^®^) and pertuzumab (PERJETA^®^) may have been used to treat HER_2^+^_ disease, according to prescribed practice during our study period. 

### 4.3. Neoadjuvant Chemotherapy (NACT)

NACT is frequently used in the treatment of locally advanced tumors such as IBC [27]. By facilitating tumor shrinkage, NACT may convert a previously unresectable cancer to an operable one [25,29,48,49,50,51]. Tumor downstaging also increases the suitability of BCS vs. mastectomy. Importantly, the efficacy of NACT can be assessed in vivo, allowing the change or discontinuation of treatment. 

### 4.4. The Importance of Acheiving pCR

Overall, clinical and radiologic findings do not correspond well with residual disease after therapy, necessitating the need for pathologic evaluation of tumor response [27]. Achieving pCR following NACT is an important surrogate endpoint of breast cancer survival, especially for high grade and aggressive cancers like HER_2^+^_ or TNBC. Increasingly, pCR is being used as a short-term endpoint in neoadjuvant clinical trials, given its prognostic association with longer-term outcomes [52].

### 4.5. Comparison with Published Studies

Our results differ from a recent analysis of patients with IBC in the SEER database, which reported the best survival outcome for HR^+^/HER_2^+^_ [35]. Approximately 20% of patients in the SEER analysis had HR^+^/HER_2^+^_ tumors compared with 36% in our study. This may be explained by different inclusion criteria and disease definition in the latter study. For example, we only included non-metastatic patients and also were able to more comprehensively identify IBC patients based on both clinical and pathologic characteristics. Additionally, patients with unknown biologic subtype were excluded in the SEER analysis. Nonetheless, this study reported poor survival for patients with TN-IBC, which is consistent with our report and other studies in the literature [24,53,54,55].

In a small single center study (*n* = 316) of newly diagnosed IBC between 1989–2008, HR^−^/HER_2^+^_ had inferior survival to HR^+^/HER_2^+^_ and HR^+^/HER_2^−^_ [54]. Again, this differs from our results which found similar survival outcomes for the above biologic subtypes. Likely, this reflects the use of HER_2^+^_ targeted therapies in our study population, whereas many of the patients in the former study preceded the introduction of this treatment option. Additionally, 99% of patients with IBC in this tertiary cancer care center received NACT, compared with ~29% in the current analysis. Even among patients in our study who did not achieve pCR, survival was better for those with HR^−^/HER_2^+^_ tumors, than other biologic subtypes. This would suggest that HER_2^+^_ targeted therapies provide systemic benefit independently of achieving pCR, targeting microscopic residual tumor. This study also had a higher percentage of patients with TN-IBC vs. the current analysis (28% vs. 10%), characteristic of their tertiary referral population. Nonetheless, the pCR rate among TN-IBC patients in both studies were similar (14% vs. 13%). Additionally, the relative percentage rankings of pCR for the remaining biologic subtypes were similar in both studies, although the absolute percentages were lower in the current study (i.e., HR^+^/HER_2^−^_ = 10%, HR^+^/HER_2^+^_ = 16%, HR^−^/HER_2^+^_ = 31% vs. HR^+^/HER_2^−^_ = 6%, HR^+^/HER_2^+^_ = 13%, HR^−^/HER_2^+^_ = 27%). 

A comparison of our findings with a recent population-based study of IBC using data from the SEER program is difficult to interpret because of design differences. Specifically, with the exception of HR^+^/HER_2^+^_, OS in our study was substantively higher than the SEER study, likely attributable to the inclusion of stage IV disease in their study. Additionally, patients were excluded if they were diagnosed without positive histology. However, this contrasts with the definition of IBC as a primarily clinical diagnosis. Patients also were excluded if they initially presented with stage III not otherwise specified disease, in comparison with the inclusion of these patients in our study [36]. Furthermore, their relatively higher percentage of TN-IBC patients likely reflects the inclusion of stage IV disease. Interestingly, the relative frequency of stage IIIb compared with stage IIIc tumors (80%) in the SEER analysis was similar to our findings (82%). 

Our pCR rates were considerably lower than a prospective cohort study of 155 registry patients from the US and Canada. While 90% of IBC patients in the latter study responded to NACT (49% complete response, and 40% partial response), this determination was based mainly on clinical features and behaviors. Women were defined as having a complete response if they reported rapid resolution to normal breast size and appearance, usually prior to the second round of chemotherapy. Similarly, partial response was defined as an incremental but definite improvement over several courses of chemotherapy, with clinical manifestations minimal or absent at the time of mastectomy. In contrast to our study, patients were self-enrolled, being required to proactively contact the registry in order to participate in the study. Additionally, patients with secondary IBC were allowed to participate in the study. Nonetheless, both studies reported a higher HR rate among women who did not respond to NACT [56].

Approximately 16% of patients in our sample were black. This compares with 17% of IBC patients in the United States Cancer Statistics (USCS) database (a population-based surveillance system of cancer registries with data representing 98% of the US population) who were black [57]. However, only 11% of IBC patients in SEER were black. The latter study also reported that 15% of TN-IBC patients were black compared with 25% in our study. Our percentages of black patients within the three other subtypes of IBC (HR^+^/HER_2^−^_ (14%), HR^+^/HER_2^+^_ (16%), and HR^−^/HER_2^+^_ (17%)) also were higher than the SEER study (HR^+^/HER_2^−^_ (12%), HR^+^/HER_2^+^_ (7%), and HR^−^/HER_2^+^_ (10%)). This concurs with other studies in literature suggesting that incidence rates of IBC are higher for black patients than their white counterparts [5,8].

In our study, as tumor size increased, the percentages of patients within each category increased in a corresponding fashion (≤2 cm, 12%; >2–5 cm, 43%; >5 cm, 46%). This pattern is consistent with a population-based study of IBC using SEER (from 2010–2013), which reported a similarly increasing percentage of patients within increasing categories of tumor size (≤2 cm, 15%; >2–5 cm, 34%; >5 cm, 50%) [35]. Furthermore, a similar pattern was observed in a linkage study of IBC (from 1998–2002) using the Florida Cancer Data System (FCDS) dataset and the Florida Agency for Health Care Administration (AHCA) database (<2 cm, 3.9%; >2–5 cm, 14.1%; >5 cm, 77.5%) [58].

After adjusting for other relevant risk factors, black race (referent = white race; HR = 2.6, *p* = 0.023) and Hispanic ethnicity (referent = white race; HR = 3.5, *p* = 0.014) were both associated with shorter survival in a comprehensive analysis of the IBC registry at MD Anderson Cancer Center [59]. In comparison, black patients in our study also were observed to have shorter survival, although the effect size was diminished (i.e., adjusted HR = 1.3). On the other hand, we observed a slightly protective effect of Hispanic ethnicity on survival (i.e., adjusted HR = 0.81). However, it should be noted that the referent group for the latter comparison in our study was non-Hispanic patients, wherein the reference group for Hispanic ethnicity in the MD Anderson study was white race. This poses a certain degree of ambiguity, as white race and Hispanic ethnicity are not mutually exclusive groups. 

Our findings for IBC were comparable with other studies of breast cancer as a whole. A significantly higher percentage of patients with HR^−^/HER_2^+^_ achieved pCR, compared with other biologic subtypes [26,52,60,61,62]. This supports the general belief that HR status is an important mechanism of underlying chemoresistance in this biologic subtype [63,64]. Additionally, achieving pCR was associated with superior OS and this was especially pronounced for patients with TN disease [28,65]. Similar to the literature, our pCR rate was found to be lower for the HR^+^/HER_2^−^_ subtype [24].

Additionally, the percentage of patients receiving NACT in our study was lower in comparison with two other studies conducted at tertiary referral centers. Such facilities often have established protocols for the treatment of IBC, which includes the use of primary systemic therapy [66,67]. Approximately 54% of patients in the current analysis had tumors ≤5 cm, while nearly 50% were treated at a community cancer center. In many cases, these patients proceeded directly to surgery. 

### 4.6. Use of Triple-Modality Therapy in IBC

In the past, using single-modality therapy including either surgery or radiation, the survival of IBC was extremely poor (~5%). Although the use of triple-modality therapy for IBC has increased in recent times, OS remains low in comparison with non-IBC [21,68]. For example, in a population-based analysis of the SEER database, the 5-year OS of patients with estrogen receptor positive (ER^+^) IBC was 49% and 25% for ER^−^ IBC. This is in comparison with 91% of women presenting with non-IBC ER^+^ tumors and 77% of those with ER^−^ tumors, respectively [68]. The peak hazard rate (53%) among women with ER^−^ tumors occurred in the 12th month following their diagnosis, compared with 8% in the 17th month for non-IBC cases. However, beyond 7-years, there were no significant differences in hazard rates between ER^−^ and ER^+^ tumors, for either IBC or non-IBC. 

In an earlier study using NCDB, the use of triple-modality therapy increased from 58% in 1998 to the highest level of 73% in 2004 [21]. On average (across biologic subtypes), RT was used in 71% of patients in the current analysis, conveying a significant survival advantage (HR = 0.63, *p* < 0.0001). This is similar to a recent study of 7304 women with non-metastatic IBC, wherein radiotherapy was associated with improved 5-year survival (adjusted HR = 0.64, 95% CI 0.61–0.69) [69]. Nonetheless, a multidisciplinary multimodal approach consisting of NACT followed by surgery and post-mastectomy radiation has been underutilized in previous reports of IBC [55,70].

### 4.7. Strengths and Limitations

Little is known about IBC especially in the context of biologic subtypes. By using NCDB, a multi-centric sample, we were able to analyze the data by these groups, while adjusting for outcome related covariates. To our knowledge, this is the first large-scale study of IBC, as defined by clinical, pathologic, histologic, and immunohistochemical characteristics. 

While NCDB is the most comprehensive collection of IBC in the US, it may underrepresent certain priority populations and those lacking comprehensive health insurance [71]. Significant variability also may exist in how data was reported across NCBD sites, limiting the generalizability of our results. Because we restricted our analysis to patients who underwent surgical resection, this sample was relatively healthy with few or no comorbidities

A uniform protocol did not exist across NCDB sites for how biologic subtypes were ascertained. For example, cut-off points for nuclear staining of invasive cells for establishing ER/PR positivity may have vary between centers. Similarly, some laboratories may have used gene amplification vs. immunohistochemical staining or fluorescence in situ hybridization to determine HER_2_ positivity. Additionally, there may have been subjectivity in how clinicians interpreted laboratory results for biologic subtype status, with some practitioners opting to administer treatment in borderline cases. For example, 45 patients with TN-IBC in our study were reported to have received endocrine therapy. 

Information on genomic profiling, functional imaging, tumor markers (e.g., EZH2, p53, CXCR4, CCR7, Notch-1, E-cadherin, VEGF-C, VEGF-D, VEGFR-3, Prox-1, lymphatic vessel endothelial receptor1, RhoC GTPase) and disease-specific survival was not available in NCDB. In particular patients with basal-like TNBC, compared with other molecular subtypes, have been reported to have improved survival after chemotherapy in breast cancer patients overall, suggesting the importance of molecular profiling [21,72]. While the NCDB does not provide details on the specific systemic therapy administered to the patients, it is reasonable to assume that most patients with HER_2^+^_ disease received targeted therapies such as Trastuzumab, as these agents became available in the early part of the last decade. This is similarly true for the increased use of anthracyclin and taxanes (e.g., paclitaxel and docetaxel) in the modern era of IBC treatment. Accordingly, our analyses provide useful information on this treated population, compared with pre-modern era studies when these compounds were not available. 

The definition of pCR varies in the literature, depending on the presence of carcinoma in situ or evidence of cancer in the axillary lymph nodes (i.e., *ypT0 ypN0; ypT0/is ypN0; ypT0/is ypN0/+; ypT ≤ 1mic ypN0/+*) [25,26,27]. Furthermore, six classification systems (i.e., AJCC, sixth edition; NSABP B-18; Miller-Payne; Chevallier; Sataloff; RCB) has been used in various clinical investigations to categorize the extent of disease present following chemotherapy [27]. Based on individual clinician documentation (as reported to the NCDB) response to NACT in our study was coded as a unique site-specific field (CSF-21), with three outcomes: (1) complete response, (2) partial response, and (3) no response. Unknown or indeterminate responses were assigned to these outcomes using the EM algorithm. While some uncertainty remained in the definition of pCR, our outcomes among patients achieving pCR were congruent with several published results showing a survival benefit [26,27,60,61]. 

Future studies will benefit by using a uniform criteria to identify IBC and incorporating information on loco-regional control. Obtaining functional phosphoproteomics data (e.g., hyperactive kinases such as (PRKCE, P70S6K, PNKP, ERK1/2, c-KIT, CDK6) also may be important when developing new prognostic models and treatment strategies for TN-IBC [73,74]. The imprecise assessment of early response to NACT and the lack of noninvasive means of predicting pCR are continuing challenges that need to be carefully addressed as investigators design new studies in this field [25].

## 5. Conclusions

This study demonstrates that the achieving pCR confers a survival benefit for all biologic subtypes of IBC. Patients with TN-IBC were observed to have the worst survival outcome overall and this was independent of pCR status. Survival also was better for HR^+^ cases, as these patients have the additional option of receiving endocrine therapy following surgery. For example, standard treatment includes Tamoxifen therapy for 5–10 years after surgery. Unfortunately, this option is not effective for TN disease.

## Figures and Tables

**Table 1:** Patient demographic characteristics for IBC (*n* = 8550, 2004–2013) ^§^.

Characteristic	Biologic Subtype				*p* Value
	HR^+^/HER_2^−^_ *n* (%) Median [IQR]	HR^+^/HER_2^+^_ *n* (%) Median [IQR]	HR^−^/HER_2^+^_ *n* (%) Median [IQR]	HR^−^/HER_2^−^_ *n* (%) Median [IQR]	
Overall *n* (%)	4005 (47)	3082 (36)	610 (7)	853 (10)	
Age (years) ^‡^ ≥50	57 [19] 2831 (71)	56 [19] 2087 (68)	56 [17] 414 (68)	56 [19] 589 (69)	0.011 ^¶^
					0.049 ^†^
Facility type Academic/research Community Comprehensive community Integrated network	1235 (31) 512 (13) 1872 (47) 387 (10)	946 (31) 345 (11) 1473 (48) 318 (10)	200 (33) 83 (14) 282 (46) 45 (7)	285 (33) 91 (11) 395 (46) 82 (10)	0.14 ^†^
Great circle distance (miles)	9 [16]	9 [16]	10 [15]	9 [15]	0.73 ^¶^
Health insurance Medicaid Medicare Other government Private None	555 (14) 1181 (29) 38 (1) 2056 (51) 175 (4)	438 (14) 822 (27) 27 (1) 1642 (53) 153 (5)	106 (17) 160 (26) 3 (<1) 308 (50) 33 (5)	142 (17) 233 (27) 8 (1) 410 (48) 60 (7)	0.0037 ^†^
Hispanic ^‡^	324 (8)	252 (8)	54 (9)	89 (10)	0.14 ^†^
Income <$38,000 $38,000–$47,999 $48,000–$62,999 $63,000+	713 (18) 943 (24) 1169 (29) 1180 (29)	564 (18) 768 (25) 863 (28) 887 (29)	116 (19) 151 (25) 184 (30) 159 (26)	202 (24) 200 (23) 238 (28) 213 (25)	0.0065 ^†^
Black race ^¥^	578 (14)	492 (16)	103 (17)	212 (25)	<0.0001 ^†^

^§^ Non-metastatic, pathologically confirmed, primary tumors. ^‡^ The reference group for binary coded variables was the complement of the indicated category. ^¥^ Other categories included ‘white race’ and ‘other race’. ^†^ Chi-square test. ^¶^ Kruskal-Wallis H test. AJCC: American Joint Committee on Cancer. HER = Human epidermal growth factor receptor. HR = Hormone receptor. IBC = Inflammatory breast cancer. IQR = Interquartile range.

**Table 2:** Patient clinical characteristics for IBC (*n* = 8550, 2004–2013) ^§^.

Characteristic	Biologic Subtype				*p* Value
	HR^+^/HER_2^−^_ *n* (%) Median [IQR]	HR^+^/HER_2^+^_ *n* (%) Median [IQR]	HR^−^/HER_2^+^_ *n* (%) Median [IQR]	HR^−^/HER_2^−^_ *n* (%) Median [IQR]	
Overall *n* (%)	4005 (47)	3082 (36)	610 (7)	853 (10)	
Clinical stage (AJCC) IIIb IIIc	3315 (83) 690 (17)	2522 (82) 560 (18)	498 (82) 112 (18)	676 (79) 177 (21)	0.12 ^†^
Charlson/Deyo score 0 1 2	3367 (84) 519 (13) 119 (3)	2573 (83) 420 (14) 89 (3)	516 (85) 78 (13) 16 (3)	700 (82) 120 (14) 33 (4)	0.67 ^†^
Differentiation (Grade) Well (I) Moderately (II) Poorly (III) Undifferentiated (IV)	161 (4) 1676 (42) 2122 (53) 46 (1)	107 (3) 1249 (41) 1676 (54) 50 (2)	5 (1) 168 (28) 420 (69) 17 (3)	4 (<1) 192 (23) 637 (75) 20 (2)	<0.0001 ^†^
Lymph node invasion ^‡^	3658 (91)	2845 (92)	523 (86)	763 (89)	<0.0001 ^†^
Margins (positive) ^‡^	558 (14)	370 (12)	56 (9)	108 (13)	0.0036 ^†^
Tumor size (cm) ^¥^ ≤2 >2–5 >5	455 (11) 1763 (44) 1787 (45)	373 (12) 1339 (43) 1370 (44)	80 (13) 248 (41) 282 (46)	88 (10) 293 (34) 472 (55)	<0.0001 ^†^

^§^ Non-metastatic, pathologically confirmed, primary tumors. ^‡^ The reference group for binary coded variables was the complement of the indicated category. ^†^ Chi-square test. ^¥^ At the time of diagnosis. AJCC: American Joint Committee on Cancer. HER = Human epidermal growth factor receptor. HR = Hormone receptor. IBC = Inflammatory breast cancer. IQR = Interquartile range.

**Table 3:** Treatment variables for IBC (*n* = 8550, 2004–2013) ^§^.

Treatment	Biologic Subtype				*p* Value
	HR^+^/HER_2^−^_ *n* (%) Median [IQR]	HR^+^/HER_2^+^_ *n* (%) Median [IQR]	HR^−^/HER_2^+^_ *n* (%) Median [IQR]	HR^−^/HER_2^−^_ *n* (%) Median [IQR]	
Overall *n* (%)	4005 (47)	3082 (36)	610 (7)	853 (10)	
Chemotherapy ^‡^	3576 (89)	2801 (91)	570 (93)	807 (95)	<0.0001 ^†^
Endocrine therapy ^‡^	2423 (61)	1591 (52)	76 (12)	45 (5)	<0.0001 ^†^
Immunotherapy (HER_2_^+^) ^‡^	NA	155 (5)	92 (15)	NA	<0.0001 ^†^
Neoadjuvant therapy ^‡^ Response NR pCR PR	1102 (28) 488 (44) 71 (6) 543 (49)	605 (20) 286 (47) 76 (13) 243 (40)	281 (46) 75 (27) 77 (27) 129 (46)	464 (54) 128 (28) 59 (13) 277 (60)	<0.0001 ^†^
					<0.0001 ^†^
Radiotherapy ^‡^ Dose (cGy) 4000–5000 >5000–6000 Lymph nodes treated ^‡^	2888 (72) 5040 [40] 1106 (38) 1782 (62) 2002 (69)	2176 (71) 5040 [40] 824 (38) 1352 (62) 1534 (71)	436 (71) 5040 [40] 163 (37) 273 (63) 302 (69)	611 (72) 5040 [40] 241 (39) 370 (61) 448 (73)	0.58 ^†^
					0.99 ^¶^
					0.89 ^†^
					0.24 ^†^
Surgery BCS/Partial mastectomy Mastectomy Contralateral ^‡^	233 (6) 3772 (94) 782 (21)	182 (6) 2900 (94) 575 (20)	28 (6) 582 (95) 143 (25)	44 (5) 809 (95) 183 (23)	0.53 ^†^
					0.041 ^†^

^§^ Non-metastatic, pathologically confirmed, primary tumors. ^‡^ The reference group for binary coded variables was the complement of the indicated category. ^†^ Chi-square test. ^¶^ Kruskal-Wallis H test. BCS = Breast conserving surgery (lumpectomy). cGy = centigray. NR = No response. HER = Human epidermal growth factor receptor. HR = Hormone receptor. IBC = Inflammatory breast cancer. IQR = Interquartile range. pCR = Pathologic complete response. PR = partial response.

**Table 4:** Multivariable Cox regression survival model (5-years) for IBC (*n* = 8550, 2004–2013) ^†^.

Characteristic ^§^	HR (95% CI)
Chemotherapy (−)	2.0 (1.8–2.2)
Endocrine therapy (−)	1.9 (1.8–2.1)
Margins (+)	1.8 (1.7–2.0)
Triple negative	1.8 (1.6–2.0)
Radiotherapy (−)	1.6 (1.5–1.7)

^†^ Variables with HR ≥ 2.0 (or ≤0.50) and *p* < 0.0001 in univariable analysis were included in the multivariable Cox regression survival model. ^§^ Pairwise adjustment for age, clinical stage, comorbidities, facility type, grade, great circle distance, Hispanic ethnicity, immunotherapy, income, insurance status, lymph node invasion, lumpectomy, neoadjuvant therapy, race, and tumor size did not substantively impact the model. CI = Confidence interval. HR = Hazard ratio. IBC = Inflammatory breast cancer.
